# Efficacy of Pomegranate Mouthrinse Compared to Chlorhexidine on Plaque Accumulation, Gingival Inflammation, and Salivary Bacterial Count: A Randomized Controlled Trial

**DOI:** 10.7759/cureus.93612

**Published:** 2025-09-30

**Authors:** Anoop Sasi, Shahana C Mohamed, Lizy Mathew, Roshni Ramesh, Raseena Beevi N

**Affiliations:** 1 Periodontology, Government Dental College, Thiruvananthapuram, Thiruvananthapuram, IND; 2 Microbiology, Government Medical College, Thiruvananthapuram, Thiruvananthapuram, IND; 3 Periodontology, Governmental Dental College, Alappuzha, Alappuzha, IND

**Keywords:** antiplaque, bacterial count, chlorhexidine, pomegranate, randomized controlled trial (rct)

## Abstract

Introduction

Dental plaque is a primary etiological factor in the development of gingivitis and periodontitis. Chlorhexidine is widely regarded as the gold standard antiplaque agent; however, its long-term use is associated with undesirable side effects, such as tooth staining, altered taste sensation, and mucosal irritation. Natural agents with antimicrobial and anti-inflammatory properties are being explored as safer alternatives. This study aimed to compare the efficacy of a 30% pomegranate peel extract (PPE) mouthrinse with 0.12% chlorhexidine (CHX) mouthwash in reducing dental plaque, gingival inflammation, and salivary bacterial count.

Methodology

A parallel-group, double-blinded, randomized controlled trial was conducted involving 60 participants aged over 18 years with baseline gingival and plaque index scores ≥1 and at least 12 gradable teeth. Participants were randomly allocated to either the experimental group (30% PPE mouthrinse) or the control group (0.12% CHX). Subjects rinsed twice daily with 10 ml of their respective mouthwash for 21 days. Plaque index (Silness and Loe) and gingival index (Loe and Silness) were assessed at baseline, 5 days, and 21 days, whereas salivary bacterial counts were assessed at baseline, half an hour after rinsing, 5 days, and 21 days. Data were analyzed using Mann-Whitney U and Wilcoxon signed-rank tests with statistical significance set at p<0.05.

Results

Both groups exhibited significant intra-group reductions in plaque index, gingival index, and bacterial counts at 5 and 21 days compared to baseline. The PPE group demonstrated a significantly greater reduction in plaque index at both 5 days (mean: 1.16 ± 0.26) and 21 days (mean: 0.79 ± 0.27) compared to the CHX group (1.40 ± 0.30 and 1.05 ± 0.29), respectively. No statistically significant intergroup differences were observed for the gingival index and long-term bacterial count. However, the PPE group showed a lower salivary bacterial count at half an hour post-rinsing (p=0.008).

Conclusion

Both 30% pomegranate and 0.12% CHX mouthrinses are effective in reducing plaque, gingival inflammation, and bacterial count over 21 days, with the PPE showing a superior short-term antiplaque effect. PPE represents a cost-effective and accessible alternative, warranting further long-term evaluation as a preventive and curative agent for oral care.

## Introduction

The antiplaque property of chlorhexidine (CHX) was discovered in the 1970s, and since then, studies in this regard have consistently confirmed CHX mouthwash as the gold standard [1]. CHX mouthwash, with a concentration of 0.1% to 0.2%, has shown efficacy even in the absence of mechanical plaque control. This gold standard also shows its effectiveness as a long-term adjunct to oral hygiene, with benefits observed at four to six weeks and up to six months of use [1]. The efficacy of CHX also depends on patients' proper compliance with its use [2]. Certain components in the toothpaste, such as calcium and anionic surfactants like sodium lauryl sulfate, sodium dodecyl sulfate, and cocamidopropyl betaine, reduce the substantivity and overall effectiveness of CHX. Thus, a 30-minute waiting time for the use of CHX mouthwash after toothbrushing is highly important [2]. When compared to other CHX formulations, CHX mouthwash with a concentration of 0.1-0.2% has been proven to be superior in antiplaque activity [1]. Thus, the efficacy of CHX is proven beyond doubt. However, the prolonged use of CHX is associated with several side effects, including tooth staining, altered taste, a burning sensation, dry mouth, and hypersensitivity [3]. The cost-effectiveness of CHX is another major concern. The higher cost of CHX compared to other mouthwashes makes it unaffordable for individuals with lower socioeconomic status [4]. It is in this regard that alternatives to CHX came into the scenario.

A systematic review on the efficacy of pomegranate showed that *Punica granatum* L. is effective in reducing plaque, destroying harmful microorganisms, and protecting oral health [5]. A randomized controlled trial in Tamil Nadu showed the effectiveness of pomegranate peel in reducing gingivitis and total bacterial count at the end of the 90-day follow-up period [6]. Pomegranate demonstrated its antiplaque effect even after 21 days [7]. Several clinical studies have also highlighted the use of pomegranate as an alternative to gels and mouthwashes, particularly in resource-limited settings [6-9]. However, the long-term effects and the clinical efficacy of pomegranate need further exploration.

Thus, this study aims to compare the efficacy of an herbal mouthwash prepared from pomegranate peel with that of CHX mouthwash in reducing plaque accumulation, improving gingival health, and lowering salivary bacterial count. While previous research established the antiplaque efficacy of pomegranate at 21 days, our study went beyond merely demonstrating the effect [7]. It directly compared 30% pomegranate peel extract (PPE) mouthrinse against 0.12% CHX mouthwash under controlled, randomized conditions, allowing for a direct assessment of their relative performance over the same 21-day period for plaque accumulation, gingival inflammation, and salivary bacterial count.

Study objectives

The primary objective of this study was to compare the efficacy of a 30% PPE mouthwash with that of a 0.12% CHX mouthwash in reducing plaque accumulation and gingival inflammation over a 21-day period. These outcomes were measured using the Plaque Index (PI) (Silness and Loe) and the Gingival Index (GI) (Loe and Silness), respectively. The secondary objective was to compare the efficacy of the mouthwashes in reducing the salivary bacterial load, assessed as colony-forming units (CFUs) on blood agar plates, over a 21-day period.

## Materials and methods

This parallel, double-blinded, randomized controlled trial aimed to compare the efficacy of a 30% PPE mouthwash (Intervention) against a 0.12% CHX mouthwash (Comparison) in reducing dental plaque, gingival inflammation, and salivary bacterial count (Outcomes) in 60 adult participants satisfying inclusion criteria (Population) over a 21-day period (Time). This study was conducted at the Department of Periodontology, Government Dental College, Thiruvananthapuram, and the Department of Microbiology, Government Medical College, Thiruvananthapuram (GMCT).

Ethical considerations

All ethical principles were maintained in the study. As a clinical trial, this study has been registered with the Clinical Trial Registry of India (CTRI) under registration number CTRI/2025/08/092528. The study obtained ethical clearance from the Institutional Ethics Committee, with the IEC number DCT/IEC/CT/24/05 dated 10/01/2024. This study was conducted in collaboration with the Department of Microbiology, GMCT. Appropriate permissions were taken for this collaboration. Furthermore, the participants were informed in detail about the study's purpose, the associated risks, and the benefits. Only those who provided informed consent were recruited to the study. The study participants were given the right to withdraw from the study at any point in time if they felt uncomfortable. Autonomy was encouraged, and confidentiality was assured.

Sample size

The sample size was determined using the formula, considering the mean difference and pooled standard deviation, with a standard normal variate at a 5% type I error (1.96) and a standard normal variate at a 20% type II error and 80% power (0.84). The minimum sample size was estimated to be 26.2, rounded off to 30 in each group.

Inclusion criteria

The inclusion criteria included those individuals aged more than 18 years with a GI score of ≥1 and a PI score of ≥1, a minimum of 12 gradable teeth, with no requirement for emergency oral prophylaxis, and willing to provide written informed consent to participate in the study and comply with the study visits.

Exclusion criteria

Individuals who were allergic to CHX or pomegranate, smokers, those with a history of using any steroid or antimicrobial agent in the past three months, individuals with existing periodontitis, and those currently taking any medication were excluded from the study.

Randomization

Block randomization with a block size of 6 was used for sequence generation. Allocation concealment was performed using the SNOSE (sequentially numbered, opaque, sealed envelope) technique. The custodian is the biostatistician who is not directly involved in the trial. Allocation cards were printed by the custodian. Inside each allocation card is an opaque envelope with carbon paper lining, numbered externally in a strict sequence generated prior. During the time of enrollment, the investigator writes the participant ID on the envelope before opening it. The allocation cards were distributed consecutively.

Preparation of PPE mouthrinse

Fresh pomegranate (*Punica granatum* L.) peels were desiccated under sunlight and subsequently dried overnight in a hot air oven at 60 °C for seven days. The dried peels were finely powdered, and the powder was subjected to extraction using a Soxhlet apparatus with distilled water as the solvent to obtain an aqueous extract. A concentration of 300 mg/ml of PPE was prepared for the mouthrinse formulation. Specifically, 18 g of PPE powder was dissolved in 60 ml of distilled water to achieve the required concentration [10].

Study procedure

Baseline data, including PI [11] and GI [12], were collected from 60 participants who satisfied the inclusion criteria. 0.5 ml of unstimulated saliva was collected in closed containers using the drooling method and was incubated in sheep blood agar. This procedure was done by the principal investigator. The participants were allocated to either of two groups: Group I (Experimental Group) or Group II (Control Group) by randomization. A 30% PPE mouthwash was administered to the experimental group (Group I) and 0.12% CHX to the control group (Group II). The participants were asked to rinse with 10 ml of the test or control mouthwash for one minute. This procedure was done by the co-investigator. The 30% pomegranate mouthwash and 0.12% CHX mouthwash were specifically formulated without any distinct flavor or taste and were further ensured to be matched in appearance, odor, and consistency. This careful preparation was crucial in maintaining the integrity of the double-blinding, ensuring participants remained unaware of their treatment allocation.

The principal investigator collected 0.5 ml of unstimulated saliva again, half an hour after participants rinsed with the given mouthwash. The participants were asked to rinse twice daily with 10 ml of the given mouthwash, half an hour after tooth brushing, for 21 days. The PI and GI were recorded, and saliva samples were collected on the 5th and 21st days in both groups. All the measurements were taken by a single examiner. Participants were reminded to use mouthwash twice daily, either through messages or phone calls. Mouthwashes were dispensed in black bottles without any flavor in order to blind the participants.

**Figure 1:**
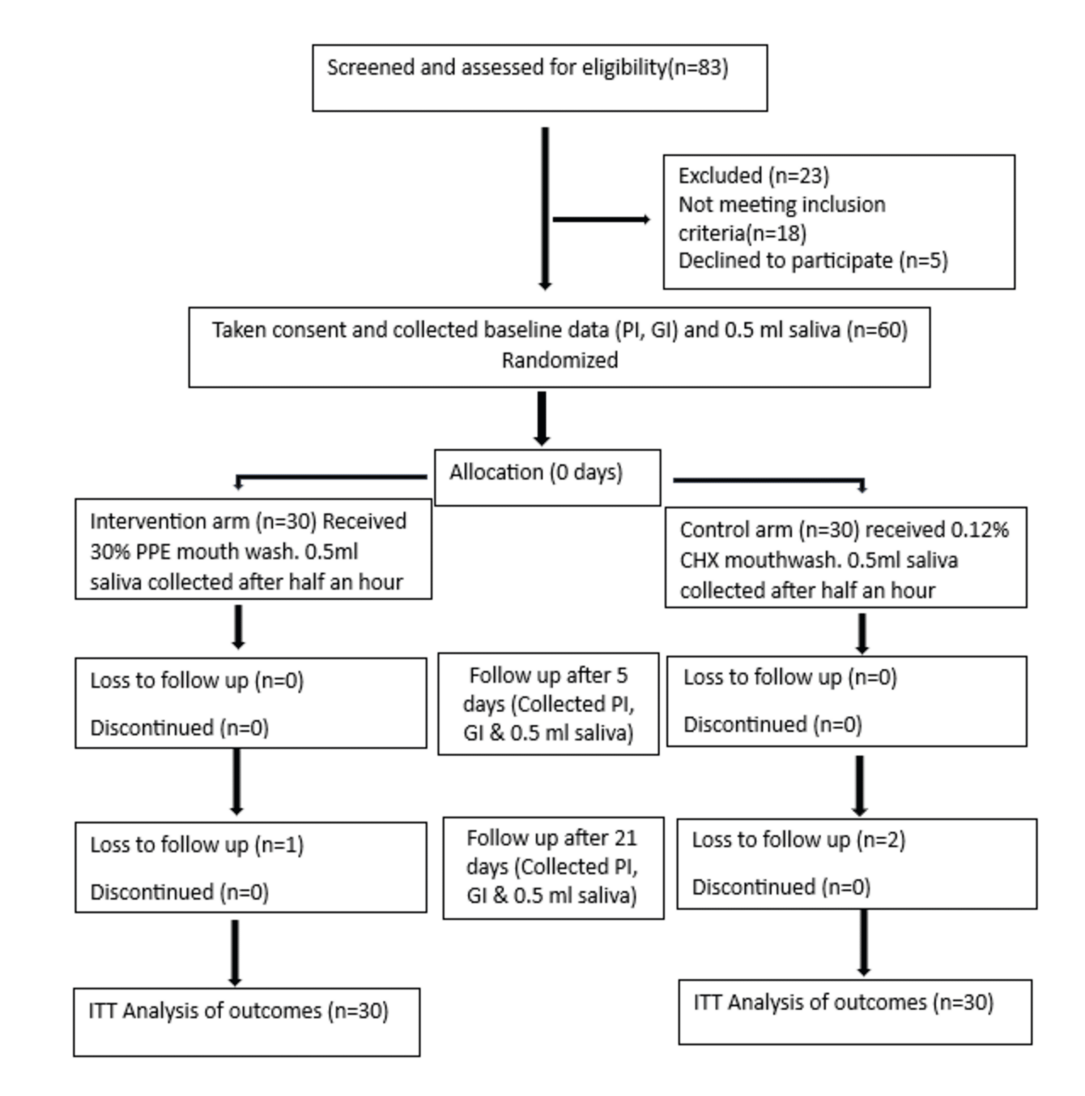
CONSORT flow diagram PPE: Pomegranate Peel Extract; CHX: Chlorhexidine; ITT: Intention-to-Treat Analysis; n: Sample Size; PI: Plaque Index; GI: Gingival Index; ml: Milliliter

Participant flow through the trial, from enrollment to final analysis, is detailed in the CONSORT flow diagram (Figure 1).

Microbiological analysis

A volume of 0.004 ml of the saliva sample was aseptically inoculated onto sterile blood agar plates. The plates were incubated at 37°C for 48 hours under aerobic conditions. Following incubation, microbial growth was assessed both quantitatively and qualitatively. The number of CFUs was determined using a digital colony counter for each plate. Colonies were subjected to morphological evaluation to characterize the isolated bacterial species. Further identification was performed by Gram staining followed by a series of standard biochemical tests (e.g., catalase, coagulase, oxidase, and carbohydrate fermentation assays) for confirmation of bacterial isolates [13].

The plaque and gingival indices of the study participants were followed up at 5 and 21 days. The qualitative and quantitative analysis of the bacteria was also followed up at half an hour, 5 days, and 21 days.

Statistical analysis

The data obtained were entered into Microsoft Excel (Microsoft Corporation, Redmond, Washington) and analyzed using IBM SPSS Statistics for Windows, Version 25 (Released 2017; IBM Corp., Armonk, New York). The primary efficacy analysis adhered to the intention-to-treat (ITT) principle, where all randomized participants were analyzed within their assigned groups, regardless of adherence to the intervention or completion of the full study period. For participants with missing outcome data, the Last Observation Carried Forward (LOCF) method was employed. To further assess the robustness of our findings and address potential departures from the missing at random assumption inherent in LOCF, a worst-case scenario sensitivity analysis was conducted, imputing missing data points based on the least favorable possible outcomes for each group to explore the impact of missing not at random mechanisms. The PI and GI scores were expressed as means and standard deviations. The bacterial count was also expressed as the mean and standard deviation, as well as the median and interquartile range. Inter- and intragroup comparisons were done. The normality of data was assessed using the Shapiro-Wilk and Kolmogorov-Smirnov tests. The data were found to be non-normal. The Wilcoxon signed-rank test was used to examine the differences in mean scores at various time intervals within a group. The Mann-Whitney U test was used to determine the differences in plaque, gingival indices, and bacterial counts among the groups. A p-value less than 0.05 was found to be statistically significant.

## Results

A total of 60 participants who met the inclusion criteria were randomly allocated into two groups: the pomegranate group (PPE) (Group I) and the CHX group (Group II). The PI, GI, and bacterial count for both groups were recorded at baseline and found to be comparable. The groups were observed for the outcome variables (PI, GI) at 5 days and 21 days.

The Silness and Loe PI showed a higher reduction in plaque score at 5 days and 21 days among Group I, and their difference was found to be statistically significant. Table 1 shows the PI scores among the two groups at follow-up periods of 5 and 21 days. An intra-group comparison of the PI shows a reduction in PI score among both groups during the follow-up periods, with the greatest reduction in score at 21 days and the highest difference in score between baseline and 21 days (Table 2).

**Table 1:** Comparison of plaque index among the groups using the Mann-Whitney U test *statistically significant. N is the sample size, the p-value is the probability value (< 0.05 is considered statistically significant), and the statistical test used is the Mann-Whitney U test.

Follow-Up	Groups	N	Mean	Standard Deviation	Mean Difference	Test Statistic	p-value
5 days	30% pomegranate	30	1.16	0.26	-0.24	3.236	0.004*
	0.12% CHX	30	1.4	0.30			
21 days	30% pomegranate	30	0.79	0.27	-0.26	3.563	0.001*
	0.12% CHX	30	1.05	0.29			

**Table 2:** Intragroup comparison of plaque index using the Wilcoxon signed-rank test *statistically significant. df is degrees of freedom, the p-value is the probability value (<0.05 is considered statistically significant), and the statistical test used is the Wilcoxon signed-rank test.

Follow-Up Periods	Mean Difference	Standard Deviation of Differences	Standard Error of Mean Difference	Test Statistic	df	p-value
30% Pomegranate						
5 to 21 days	0.36	0.23	0.04	8.554	29	0.000*
Baseline to 5 days	0.73	0.15	0.03	27.280	29	0.000*
Baseline to 21 days	1.09	0.34	0.06	17.865	29	0.000*
0.12% CHX						
5 to 21 days	0.34	0.15	0.03	12.526	29	0.000*
Baseline to 5 days	0.61	0.23	0.04	14.603	29	0.000*
Baseline to 21 days	0.96	0.34	0.06	15.548	29	0.000*

A comparison of the Loe and Silness GIs did not show a significant difference between the two groups. The mean GI at 5 days and 21 days in the pomegranate group was 1.32±0.29 and 1.03±0.27, respectively, and in the CHX group, it was 1.29±0.34 and 1.07±0.3, respectively. The GI score at 5 days was found to be lower in the CHX group, and at 21 days in the pomegranate group; however, the difference between these groups is not statistically significant. An intragroup comparison of GI scores revealed a higher mean difference at different times in the pomegranate group compared to the CHX group. An intragroup comparison revealed statistically significant differences between the different time periods in both groups (Table 3).

**Table 3:** Intragroup comparison of the gingival index using the Wilcoxon signed-rank test *statistically significant. df is the degrees of freedom, the p-value is the probability value (<0.05 is considered statistically significant), and the statistical test used is the Wilcoxon signed-rank test.

Follow-Up Periods	Mean Difference	Standard Deviation of Differences	Standard Error of Mean Difference	Test Statistic	df	p-value
30% Pomegranate						
5 to 21 days	0.30	0.16	0.03	10.106	29	0.000*
Baseline to 5 days	0.72	0.23	0.04	17.410	29	0.000*
Baseline to 21 days	1.02	0.25	0.04	22.722	29	0.000*
0.12% CHX						
5 to 21 days	0.22	0.15	0.03	7.740	29	0.000*
Baseline to 5 days	0.60	0.17	0.03	19.083	29	0.000*
Baseline to 21 days	0.82	0.19	0.04	23.404	29	0.000*

Apart from the baseline, bacterial counts were also estimated at half an hour, 5 days, and 21 days. The pomegranate group showed a slightly lower bacterial count at the follow-up period compared to the CHX group, but the difference at both 5 days and 21 days was not statistically significant. A statistically significant difference was observed in the half-hour bacterial count among the groups, with a lower bacterial count in the pomegranate group (Table 4).

**Table 4:** Intergroup comparison of bacterial count using the Mann-Whitney U test *statistically significant. N is the sample size, CFU is colony-forming units, p-value is the probability value (<0.05 is considered statistically significant), and the statistical test used is the Mann-Whitney U test.

Follow-Up	Groups	N	Mean (CFU)	Standard Deviation	Mean Difference	Test Statistic	p-value
Half an hour	30% pomegranate	30	28.20	10.324	-2.33	0.631	0.008*
	0.12% CHX	30	30.53	17.423			
5 days	30% pomegranate	30	24.87	7.749	-1.77	0.671	0.204
	0.12% CHX	30	26.63	12.119			
21 days	30% pomegranate	30	21.17	7.742	-0.8	0.339	0.657
	0.12% CHX	30	21.97	10.374			

Table 5 presents the intragroup comparison of bacterial counts in both groups, and the mean difference was found to be higher in the pomegranate group, except for the bacterial count difference between the 5th and 21st days.

**Table 5:** Intragroup comparison of bacterial count using the Wilcoxon signed-rank test *statistically significant. df is the degrees of freedom, CFU is colony-forming units, the p-value is the probability value (<0.05 is considered statistically significant), and the statistical test used is the Wilcoxon signed-rank test.

Follow-Up Periods	Mean Difference	Standard Deviation of Mean Differences	Standard Error of Mean Difference	Test Statistic	df	p-value
30% Pomegranate						
Baseline to half an hour	39.2	15.79	2.88	13.597	29	0.000*
Baseline to 5 days	42.53	18.87	3.44	12.349	29	0.000*
Baseline to 21 days	46.23	18.8	3.43	13.471	29	0.000*
Half an hour to 5 days	3.33	8.37	1.53	2.182	29	0.037*
Half an hour to 21 days	7.03	9.75	1.78	3.951	29	0.000*
5 days to 21 days	3.7	5.84	1.07	3.468	29	0.002*
0.12% CHX						
Baseline to half an hour	37.27	19.03	3.48	10.724	29	0.000*
Baseline to 5 days	41.17	21.2	3.87	10.634	29	0.000*
Baseline to 21 days	45.83	23.7	4.33	10.591	29	0.000*
Half an hour to 5 days	3.9	8.31	1.52	2.572	29	0.016*
Half an hour to 21 days	8.57	12.1	2.21	3.878	29	0.001*
5 days to 21 days	4.67	7.64	1.39	3.348	29	0.002*

Gram-positive cocci (GPC), aerobic spore-bearing (ASB), Gram-negative cocci (GNC), Gram-negative bacillus (GNB), and diphtheria (DIPH) were estimated in each group at baseline, half an hour, 5 days, and 21 days.

A significant difference was observed between the groups for GPC at various periods, with a slightly higher value for the pomegranate group (p = 0.02). ASB bacteria showed a significant difference at 21 days among the groups, with a higher count in the CHX group (p = 0.004). GNB and DIPH did not show any significant difference between the groups. GNC showed significant differences at half an hour (p = 0.003), 5 days (p = 0.03), and 21 days (p = 0.04) among the groups at different time points, with a higher count in the CHX group.

Sensitivity analysis

To assess the robustness of our primary efficacy findings to the chosen method of handling missing data, a sensitivity analysis was conducted for the primary outcome of PI reduction. The primary ITT analysis indicated a statistically significant reduction in PI at 21 days in the 30% PPE mouthwash group compared to the 0.12% CHX group (mean difference: -0.26, p = 0.001). The 'worst-case' scenario analysis for PI at 21 days still indicated a statistically significant reduction favoring the PPE group, although with an attenuated effect size (mean difference: -0.21, p = 0.006). Conversely, the 'best-case' scenario further strengthened the observed difference favoring the PPE group (mean difference: -0.3 and p = 0.000). These findings provide further support for the robustness of the study's conclusions, despite the presence of missing data.

## Discussion

This study is relevant and justified owing to the adverse effects and costs of CHX mouthwash. However, the results show that pomegranate mouthwash is effective in reducing plaque. A significant difference in bacterial count was observed at half an hour, with a lower bacterial count in the pomegranate group. However, the bacterial count at the follow-up periods of 5 days and 21 days did not show any significant difference. Thus, the long-term effect of pomegranate in reducing the bacterial count is questionable. The GI scores did not show superior effects for pomegranate mouthwash.

However, both 30% pomegranate and 0.12% CHX were found to be effective in reducing the plaque, gingival inflammation, and bacterial count from baseline to 21 days. Mouthwash extracted from pomegranate peel is cost-effective compared to CHX in treating mild to moderate periodontitis. Being a readily available fruit, the costs mainly lie in laboratory expenses, which are less than those associated with the chemical manufacturing of CHX [14].

The primary outcome of PI reduction revealed that PPE mouthrinse performed comparably to, and in certain measures slightly better than, CHX mouthwash over both short (5 days) and extended (21 days) intervals, with statistically significant intergroup differences favoring PPE at both follow-ups. In contrast, while both groups demonstrated marked reductions in GI and salivary bacterial counts from baseline through the follow-up periods, the intergroup differences for GI and bacterial counts (except at half an hour post-rinsing) did not attain statistical significance. These findings strongly suggest that while pomegranate rinse is at least as effective as CHX in addressing plaque and gingival health, its antibacterial effect may be comparable but not superior to the gold standard in sustained use.

These results resonate with a growing body of literature asserting the potential of herbal oral rinses, including pomegranate, as viable alternatives or adjuncts to traditional chemotherapeutic agents, such as CHX, especially in resource-limited settings. A landmark systematic review established that *Punica granatum* demonstrates robust anti-plaque, antimicrobial, and anti-inflammatory properties in various oral health contexts [5]. Malhotra et al. (2019) observed similar reductions in plaque and gingival parameters over a three-month period with pomegranate peel use, corroborating the present study's results regarding both the short- and medium-term efficacy of PPE [15]. Similarly, other studies have demonstrated significant decreases in bacterial viability and plaque scores in several herbal mouthrinses, paralleling the effects of CHX [6,10,16,17].

However, some discrepancies with earlier studies are prominent. The absence of a statistically significant difference in long-term (21-day) bacterial count between the PPE and CHX groups in the present trial contrasts with the findings of Gupta et al., who reported that sustained bacterial count reduction was more pronounced in the herbal groups [18]. Such variation may arise from differing concentrations of PPE, rinsing durations, or participant population oral hygiene baselines.

The efficacy of pomegranate mouthrinse is attributed to its high content of polyphenols, flavonoids, and tannins, which exhibit documented antimicrobial, anti-inflammatory, and antioxidant activities [19]. These phytochemicals disrupt bacterial cell walls, inhibit biofilm formation, neutralize reactive oxygen species, and downregulate pro-inflammatory cytokine expression, thereby mimicking or supplementing the broad-spectrum bacteriostatic and bactericidal effects of CHX [19,20]. In this study, the rapid reduction in bacterial count by PPE at half an hour post-rinsing may result from these potent biochemical actions. However, the observed attenuation in sustained antimicrobial effect indicates the possibility of reduced substantivity and shorter intramucosal retention compared to CHX, whose cationic properties confer prolonged adherence to oral tissues.

The observed non-inferiority of PPE to CHX for controlling plaque and gingivitis dovetails with calls for safer, cost-effective, and culturally acceptable alternatives to synthetic mouthwashes. CHX, while highly efficacious, is marred by adverse effects such as tooth staining, taste alteration, and oral mucosal irritation, as well as higher costs, limiting its utility among certain populations. Herbal extracts, such as PPE, prepared from abundantly available and inexpensive raw materials, could democratize preventive oral care, particularly in low-resource or rural settings.

Strengths and limitations

The strengths of this study include rigorous blinding and randomization. The use of double-blinding, allocation concealment (SNOSE), and involvement of a biostatistician ensured minimization of selection and assessment bias. The implementation of clear eligibility criteria promoted cohort homogeneity and reduced confounding from systemic or behavioral factors. The use of standardized data collection tools (Loe and Silness GI, Silness and Loe PI) by a single examiner reduced measurement error and observer bias. Quantitative and qualitative assessments of salivary bacterial load, using conventional microbiological methods, permitted a comprehensive evaluation of antimicrobial action. Groups were well-matched at baseline, enhancing the legitimacy of observed intergroup differences. Furthermore, the novelty and scientific basis of our intervention are significantly enhanced by our unique approach to the concentration of PPE mouthrinse. Unlike many previous studies that explored varying or higher concentrations of pomegranate extracts [6,7,17], the 30% PPE mouthrinse prepared in our study was specifically formulated at a concentration of 300 mg/ml, which was inferred from previous in vitro studies to be close to or at its Minimum Inhibitory Concentration (MIC) [10]. This allowed us to specifically assess its in vivo effects at a clinically relevant antimicrobial threshold, providing a direct evaluation of its efficacy at a potent, yet potentially less concentrated, level compared to other formulations.

The limitation of this study is the short follow-up period. The study assessed changes over 21 days, which, while adequate for acute effects, does not capture long-term compliance, recurrent bacterial colonization, or chronic safety. This study was conducted in a single tertiary care institution, thus limiting its generalizability across diverse populations or geographic settings. Although participants were monitored, the objective recording or grading of adverse effects, such as staining, mucosal reactions, or taste disturbances, was limited. Owing to the budget constraints, molecular characterization or detailed profiling of the oral microbiome was not undertaken and was limited to Gram staining and selected biochemical tests. Although blinding was implemented, dietary habits, individual oral hygiene routines, and environmental variables may still have influenced the outcomes. While our findings suggest a potential cost-effectiveness advantage for PPE due to its natural origin and simpler preparation, a formal, quantitative economic evaluation encompassing all direct and indirect costs was beyond the scope of this clinical trial and constitutes a recognized limitation.

## Conclusions

This study highlights the potential of PPE as a more accessible and likely cost-effective alternative, due to its natural origin and simpler preparation, or as an adjunct to traditional gold standard rinses in preventive and curative oral care. With growing interest in integrative and herbal medicine, high-quality clinical trials such as this not only broaden evidence-based practice but also facilitate patient-centric, resource-appropriate oral health strategies for diverse communities.

To validate and generalize the findings of this study, multicenter studies with larger sample sizes and longer follow-ups are required. More molecular-based techniques should be explored further to define the precise antimicrobial modes of action and substantivity profiles of PPE compared to CHX. Furthermore, formal health economic analysis studies comparing total direct and indirect costs associated with herbal versus synthetic mouthrinses are essential to fully evaluate the cost-effectiveness of PPE. While this study focused on common oral commensals and pathogens, the spectrum of efficacy against periodontal pathogens warrants investigation. Further studies on chronic toxicity, possible allergic reactions, and long-term oral or systemic health implications of repeated PPE use are also recommended.
